# Enhancement of periodontal tissue regeneration by transplantation of osteoprotegerin-engineered periodontal ligament stem cells

**DOI:** 10.1186/s13287-015-0023-3

**Published:** 2015-03-12

**Authors:** Fang Su, Shi-Sen Liu, Jun-Li Ma, Dong-Sheng Wang, Ling-Ling E, Hong-Chen Liu

**Affiliations:** Institute of Stomatology, General Hospital of Chinese PLA, No. 28, Fuxing Road, Haidian District, Beijing, 100853 China; Department of Stomatology, The 306th Hospital of Chinese PLA, No.9 Anxiang Beili, Chaoyang District, Beijing, 100101 China; Department of Stomatology, Navy General Hospital of Chinese PLA, No. 6 Fucheng Road, Haidian District, Beijing, 100048 China

## Abstract

**Introduction:**

The objective of the present study was to evaluate the capacity of a tissue-engineered complex of human osteoprotegerin (hOPG)-transfected periodontal ligament stem cells (PDLSCs) seeding on beta-tricalcium phosphate (β-TCP) to regenerate alveolar bone defects in New Zealand rabbits.

**Methods:**

PDLSCs were isolated from rabbit periodontal ligament tissues and expanded *in vitro* to enrich PDLSC numbers, and their proliferative activities and differentiation capability were evaluated under specific induction conditions. Lentiviral vector containing hOPG and enhanced green fluorescent protein (EGFP) was constructed by using Gateway technology and transfected into rabbit PDLSCs. The expression of hOPG was determined with quantitative real-time reverse transcription-polymerase chain reaction and Western blot. The PDLSCs with or without engineered hOPG were seeded on β-TCP scaffolds prior to transplantation. Morphological characterization of cells and materials was done by scanning electron microscope. Twenty rabbits with alveolar bone defects were randomly allocated into four groups and transplanted with β-TCP, PDLSCs/β-TCP, and hOPG-transfected PDLSCs/β-TCP or were left untreated as a control. Animals were sacrificed 12 weeks after operation for histological observation and histomorphometric analysis.

**Results:**

PDLSCs expressed STRO-1 and vementin and favored osteogenesis and adipogenesis in conditioned media. Expressions of hOPG were significantly upregulated after transfection of the lentiviral vector into PDLSCs. PDLSCs attached and spread well on β-TCP, and there was no significant difference in growth of PDLSCs on β-TCP between the hOPG transfection group and the non-transfection group. The histological observation and histomorphometric analysis showed that the hOPG-transfected PDLSCs/β-TCP complex exhibited an earlier mineralization and more bone formation inside the scaffold than control, β-TCP, and PDLSCs/β-TCP complexes. Implantation of hOPG-transfected PDLSCs contributed to new bone formation as determined by EGFP gene expression under circularly polarized light microscopy.

**Conclusions:**

The present study demonstrated the feasibility of β-TCP scaffolds for primary PDLSC culture and expression of hOPG gene *in vitro* and *in vivo*, and hOPG-transfected PDLSCs could serve as a potential cell source for periodontal bone regeneration, which may shed light on the potential of systemic hOPG gene therapy in combination with PDLSC tissue engineering as a good candidate in periodontal tissue engineering for alveolar bone regeneration.

## Introduction

Periodontal disease, one of the most prevalent chronic infections in humans, is a highly prevalent chronic inflammatory condition involving bacterial infection of tooth-supporting tissues, which in turn lead to chronic inflammation and loss of teeth [1]. It has been reported to be the most common cause of tooth loss in adults, affecting about 90% of the world population [2]. Furthermore, recent evidence has established that periodontal disease is associated with several systemic conditions, including diabetes, cardiovascular disease, stroke, respiratory infections, and adverse pregnancy outcomes [3,4]. Therefore, careful treatment of periodontal disease may be of major importance for oral health as well as improvements of long-term outcome of the patients.

Various approaches have been developed to restore the structure and function of destroyed periodontium, the final goal of periodontal therapy, and treatments, including bone grafting, guided tissue regeneration, and enamel matrix derivatives, have already been approved for clinical use. However, complete regeneration is rarely accomplished by these methods [5].

Progress in tissue engineering has offered a new option to supplement existing treatment regimens for periodontal disease. Furthermore, the discovery of progenitor/stem cells residing in the periodontium raises the possibility of restoring damaged periodontal tissues by recruiting their latent regenerative potential. Owing to the difficulties encountered in isolating specialized cells and the associated morbidity involved, stem cells serve as a better alternative. Research in tissue engineering has shown the therapeutic advantages of delivering stem cells and growth factors in biodegradable scaffolds, which supply the necessary environment to recreate a suitable niche for cellular proliferation and differentiation [6].

Beta-tricalcium phosphate (β-TCP), one of the earliest calcium compounds used as a bone graft substitute, has been shown to have good biocompatibility and osteoconductivity in both animal and clinical studies [7,8], although the ectopic bone formation following β-TCP without bone marrow stromal cells or osteoinductive cytokine implantation has been demonstrated in dogs [9]. However, no bone formation was observed following β-TCP implantation into the rat subcutis, and new bone formation at scaffolds was suggested to depend partially on the potential of cells in the grafted site [10].

Periodontal ligament stem cells (PDLSCs) with characteristics of putative mesenchymal stem cells (MSCs) represent a promising cell-based therapy in reconstructive dentistry for the treatment of periodontal disease [11]. It can be isolated from periodontal ligament (PDL) cells, providing a unique reservoir of stem cells from an accessible tissue resource. Once isolated, PDLSCs can be expanded sufficiently *in vitro* and the complex differentiation processes involved in periodontal regeneration can be optimized in the right location. The transplantation of *ex vivo*-expanded PDLSCs was suggested to hold promise as a therapeutic approach for the reconstruction of tissues destroyed by periodontal diseases [12]. The use of autologous PDLSCs to treat periodontal defects in animal models of periodontitis has further demonstrated the feasibility of using PDLSC-mediated tissue engineering to treat periodontal diseases [13].

Osteoprotegerin (OPG), a member of the tumor necrosis factor (TNF) receptor superfamily of proteins, is a soluble decoy receptor for RANKL (receptor activator of nuclear factor-kappa-B ligand), a critical osteoclast differentiation factor, and thus prevents binding of RANKL to RANK and subsequent activation of osteoclast activity [14]. PDL cells were found to express both RANKL and OPG mRNA and were suggested to regulate osteoclastogenesis by opposing mechanisms—stimulation of resorptive activity by RANKL and inhibition by OPG—thus affecting processes such as periodontitis and orthodontic tooth movement [15]. The ratio of RANKL to OPG concentration was demonstrated to be significantly higher for patients with periodontal disease than for healthy subjects [16]. OPG gene transfer to periodontal tissue inhibited osteoclastogenesis and alveolar bone resorption in lipopolysaccharide-induced experimental periodontal disease [17]. Administration of kaliotoxin, a potassium channel blocker, markedly decreased the ratio of RANKL and OPG protein expression and induction of RANKL-dependent osteoclastogenesis by the activated T cells *in vitro* [18]. *In vivo* inhibition of OPG ligand function with the decoy receptor OPG diminished alveolar bone destruction and reduces the number of periodontal osteoclasts after microbial challenge [19]. All of these results identified OPG as a potential therapeutic target for periodontal disease.

Cells, scaffolds, and growth factors are the three main factors for creating a tissue-engineered construct, and incorporation of DNA into tissue-engineering matrices and its subsequent sustained release may provide an optimal means to engineer tissues [20]. The biomaterial-based gene transfer method that combines gene therapy and tissue engineering to promote tissue regeneration has been developed. Periodontal tissue engineering using *ex vivo* gene transfer has been reported to offer a safe new approach for repairing periodontal defects [21]. The objective of this study was to evaluate human OPG (hOPG) gene-engineered rabbit PDLSCs seeding on β-TCP scaffolds as prospective candidates for periodontal tissue engineering. PDLSCs were isolated from rabbit PDL cells, and their immunophenotype and multipotent capacity to differentiate into adipocytes, osteoblast-like cells, were characterized *in vitro*. Lentiviral vector containing hOPG and enhanced green fluorescent protein (EGFP) was constructed by using Gateway technology. The expression of hOPG was detected with quantitative real-time reverse transcription-polymerase chain reaction (Q-PCR) and Western blot. The β-TCP scaffold combined with virus encoding hOPG gene was constructed and evaluated for cytocompatibility through seeding rabbit peripheral and limbal corneal stromal cells (PLCSCs) into scaffold *in vitro*. Morphological characterization of cells and materials was evaluated by scanning electron microscope (SEM). Furthermore, hOPG-transfected PLCSCs-βTCP scaffolds were implanted into alveolar bone defects of rabbits to evaluate regeneration of alveolar bone defects *in vivo*.

## Methods

### Animals

This study was reviewed and approved by the Animal Ethics Committee of the People’s Liberation Army General Hospital. Healthy male New Zealand white rabbits, weighing 2.0 to 2.5 kg, were obtained from the experimental animal center of our hospital. Animals were maintained under conventional conditions with free access to food and water. All surgical procedures and care administered to the animals were approved by the Animal Care Committee and performed in accordance with institutional guidelines.

### Periodontal ligament stem cell cultures

#### Isolation and cultivation of the rabbit periodontal ligament stem cells

Rabbits used for isolating PDLSCs were killed after anaesthesia by the air embolism method. After the extraction of disease-free impacted teeth within 2 hours of death, the surfaces of the teeth were cleaned with 75% alcohol and PDL cells were gently scraped from the middle third of the root surface by using forceps and then digested in a solution of 3 mg/mL collagenase type I (Sigma Chemical, St. Louis, MO, USA) and 4 mg⁄mL of dispase (Sigma-Aldrich, St. Louis, MO, USA) for 2 hours at 37°C. Cells were dispersed into a six-well plate and incubated in the lower sugar Dulbecco’s modified Eagle’s medium (DMEM) (Gibco BRL, Grand Island, NY, USA) supplemented with 10% fasting blood sugar (FBS) (Gibco BRL) for 3 days. The cultures were kept in an incubator at 37°C and 5% CO_2_ with media changes three times per week. When 70% to 80% confluence was reached, adherent cells were detached with trypsin-EDTA (0.25%) and passaged into fresh culture flasks at a ratio of 1:3. To obtain homogeneous populations of PDLSCs, single-cell-derived colony cultures were obtained by using a limiting dilution technique.

#### MTT assay

Proliferative activities of rabbit PDLSCs were assessed by using the MTT (3-(4,5-dimethylthiazol-2-Yl)-2,5-diphenyltetrazolium bromide) assay as described previously [22]. Briefly, PDLSCs were seeded in 96-well plates at a density of 2 × 10^3^ cells per well and cultured for 9 days. Twenty microliters of MTT solution (5 g/L) was added to 10 of 90 wells each day, and the plates were incubated for 4 hours at 37°C. Then, the medium was replaced with 150 μL of dimethyl sulfoxide, and the absorbance was measured at 490 nm by an enzyme-linked immune detector.

#### Immunofluorescence staining

PDLSC identity was confirmed by immunocytochemical staining by using the mouse antibodies against rabbit vimentin and keratin (Boster Biological Technology Ltd., Wuhan, China) as well as human STRO-1 (R&D Systems, Inc., Minneapolis, MN, USA) at room temperature for 2 hours. Cells were washed with phosphate-buffered saline (PBS) and incubated with the fluorescein isothiocyanate-conjugated anti-mouse IgG (1:100; Jackson ImmunoResearch Laboratories, Inc., West Grove, PA, USA) for 1 hour at 37°C and then counterstained with the nuclear dye 4′,6-diamidino-2-phenylindole (DAPI) (Biotium, Hayward, CA, USA). The cells were rinsed three times with PBS and analyzed by using a DMIL fluorescent-inverted phase-contrast microscope equipped with a Leica MPS-30 camera (Leica, Bensheim, Germany).

#### Multipotent differentiation

To investigate the multipotency of PDLSCs, the ability for isolated cells to undergo osteogenic and adipogenic differentiation was tested. Multilineage differentiation assays toward osteogenic and adipogenic pathways were performed as previously reported [23]. To detect osteogenic differentiation, alkaline phosphatase (ALP) activity was measured 3 and 7 days after seeding, in accordance with the recommendations of the manufacturer. At the 21st day of differentiation, immunohistochemistry for type I collagen and osteocalcin was performed on the osteogenic stem cells prepared on chamber slides fixed with 10% formalin. Calcium accumulation was detected by staining with 2% Alizarin red S (Sigma-Aldrich) (pH 4.2) on post-induction day 28. Adipogenesis was assessed with Oil red O (Sigma Chemical) staining.

### Constructs and cell transfection

#### Oligonucleotide design

A genomic sequence containing hOPG cDNA was synthesized by Invitrogen Life Technologies Inc. (Gaithersburg, MD, USA), according to the GenBank accession number NM_002546, and cloned into intermediate vector containing pIRES-EGFP by using primers SF-F1 and SF-R1. The flanking region of the primers (SF-F2 and SF-R2) was constructed in accordance with the Gateway Cloning Manual (Invitrogen, Carlsbad, CA, USA), as summarized in Table 1.

**Table 1:** **Primers used for Gateway cloning of human osteoprotegerin into the lentiviral vector**

**Primer**	**Sequence (5′ → 3′)**
SF-F1	AAATAGATCTGCCACCATGAACAACTTGCT
SF-R1	ATACGTCGACAGCTGGGTCTTATAAGCAGC
SF-F2	GGGGACAAGTTTGTACAAAAAAGCAGGCTTCGCCGCCACCATGAACAACTTGCTGTGCTGCG
SF-R2	GGGGACCACTTTGTACAAGAAAGCTGGGTCTCACTTGTACAGCTCATCCATGCCG

#### Gateway cloning

Construct sequences (hOPG-pIRES-EGFP) were confirmed by sequencing and then amplified by using primers SF-F2/SF-R2 for BP reaction, which generated pDONR-hOPG-pIRES-EGFP, the entry clone in pDONR™ 221 vector. BP reaction was followed by LR reaction in accordance with the instructions of the vendor. Entry vector pDONR-hOPG-pIRES-EGFP was transferred to lentivirus expression vector pLenti6.3/V5-DEST to generate pLenti6.3/V5-hOPG-pIRES-EGFP, the final expression clone containing the N-terminal hOPG fusion. Both the entry and expression clones were confirmed by automated DNA sequencing analysis (ABI 3730 DNA analyzer; Applied Biosystems, Carlsbad, CA, USA).

#### Transfection into HEK293T and periodontal ligament stem cells

The pLENT6.3/V5-hOPG-IRES-EGFP was firstly transfected into the packaging cells HEK293T (Shanghai Yingjun Bioengineering Co., Shanghai, China) by using Lipofectamine2000 (Invitrogen) in accordance with the instructions of the manufacturer. Infectious lentiviruses were harvested after 48 hours, centrifuged to eliminate cell debris, and filtered through 0.45-μm filters. The viral titres were detected at 1.5 × 10^6^ TU/mL by infecting 293 T cells with serial dilutions of concentrated lentivirus. Subsequently, the virus supernatants from infected 293 T-cell cultures were used to infect proliferating PDLSCs at an optimized multiplicity of infection of 10 with polybrene (Sigma-Aldrich) at a final concentration of 8.0 μg/mL, and the efficiency of transfection was determined by using a fluorescent-inverted phase-contrast microscope (Olympus, Tokyo, Japan).

#### Quantitative real-time reverse transcription-polymerase chain reaction and Western blot

Two days after infection, the relative mRNA and protein levels of hOPG were determined by Q-PCR and Western blot. Total RNA was extracted by using the TRIzol reagent (Gibco BRL). First-strand cDNA was synthesized by using the SuperScript III First-Strand Synthesis System (Invitrogen). Quantification of gene expression was carried out by using Platinum Taq DNA Polymerase (Invitrogen, São Paulo, Brazil). Protein hOPG quantification with Western blot was performed as previously described [24]. All experiments were performed in triplicate, and the mean value was recorded.

### Transplantation of human osteoprotegerin-engineered periodontal ligament stem cells

#### Seeding of beta-tricalcium phosphate

β-TCP (Shanghai Beiaolu Bio-Materials Ltd.) was prepared before cell seeding as previously reported [10]. The PDLSCs with or without hOPG-containing lentiviral vector transfection were collected via trypsinization and centrifugation. The resuspended cells (5 × 10^6^/mL) were then seeded separately into sterile, resorbable β-TCP scaffolds. Grafts were incubated for 2 hours at 37°C, allowing the cells to adhere to β-TCP. After this incubation period, cells were incubated in DMEM with 10% FBS at 37°C and 5% CO_2_. Morphological characterization of cells and materials was performed by using a Hitachi S-520 SEM (Hitachi, Tokyo, Japan).

#### Generation of alveolar bone defect model

**Figure 1:**
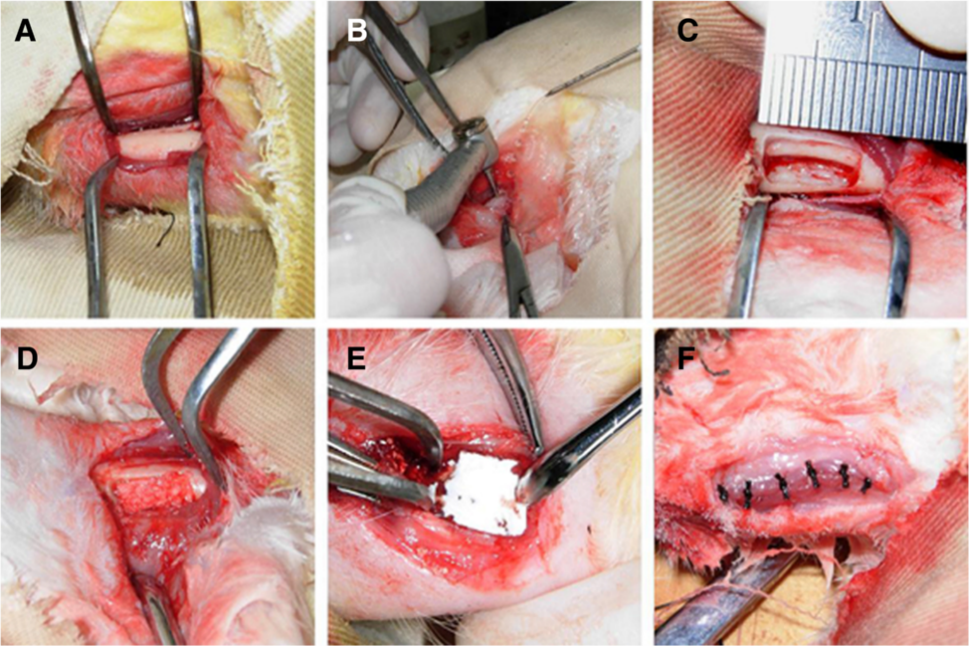
**Schematic illustration of the generation of alveolar bone defects and transplantation of the tissue-engineered complex. (A)** The exposed rabbit alveolar bone. **(B)** Bone defects using a high-speed surgical bur supplemented with copious sterile saline water irrigation. **(C)** The defect (10 × 5 × 4 mm) prepared in alveolar bone. **(D)** The tissue-engineered bone complex combined with or without periodontal ligament stem cells inside alveolar bone defect. **(E)** Collagen membranes were placed to cover the implants and defects. **(F)** The defects were closed with sutures.

Twenty male New Zealand white rabbits were used to generate a segmental critical-size alveolar bone defect model as described previously [25]. All surgical procedures were performed under general anesthesia with a combination of sumianxin II (0.25 mL/kg) and pentobarbital sodium (18 mg/kg) injected intramuscularly. A 10-mm incision was made, and the tissue overlying the diaphysis of the left alveolar bone of incisors of rabbits was dissected. A high-speed surgical bur, with copious irrigation of sterile saline, was used during preparation of the bony defects. The created alveolar bone defect was 5 mm in width, 10 mm in length, and 4 mm in depth (Figure 1).

#### Transplantation

After model generation, the defective samples in the models were randomly assigned to four different treatment groups (n = 5): (1) control group, no treatment; (2) β-TCP group, transplantation of β-TCP scaffolds; (3) PDLSCs/β-TCP group, transplantation of PDLSCs/β-TCP constructs; and (4) hOPG-PDLSCs/β-TCP group, transplantation of hOPG-transfected PDLSCs/β-TCP constructs. All of the defects were covered with gelatin membranes (Bio-Gide; Geistlich Biomaterials, Wolhusen, Switzerland) and closed with sutures. All procedures were carried out under aseptic conditions, and antibiotic was given to each rabbit for 3 days after the surgery.

#### Toluidine blue staining

At 12 weeks after transplantation, all animals were sacrificed and the mandibles were harvested. Periodontal tissue samples were prepared by using the sawing-grinding method as described by [26]. The blocks were trimmed by using an exakt grinding machine (ExaktApparatebau, Norderstedt, Germany), ground to a final thickness of approximately 20 μm, and stained with Toluidine blue, and the results were visualized by light microscopy.

#### Laser confocal microscope

Periodontal tissue samples from the hOPG-PDLSCs/β-TCP group were harvested, embedded in paraformaldehyde (4%) for 2 days, and prepared as described above. The confocal images were obtained by using a laser confocal microscope system (TCS SP2; Leica).

#### Histomorphometric analysis

For morphometric analysis, three sequential sections per implant were selected for evaluation. A histometric software package with image-capturing capabilities (Image-Pro Plus 6.0; Media Cybernetics Inc., Bethesda, MD, USA) was used to evaluate the bone formation in defects. The ratios between the regenerated bone area and the total defect area of the images were calculated.

### Statistical analysis

All statistical analyses were performed with SPSS version 17.0 (SPSS Inc., Chicago, IL, USA). Data are expressed as mean ± standard deviation. The significance of differences between groups was evaluated by one-way analysis of variance. A *P* value of less than 0.05 was considered statistically significant.

## Results

### Culture and colony efficiency assays of periodontal ligament cells

**Figure 2:**
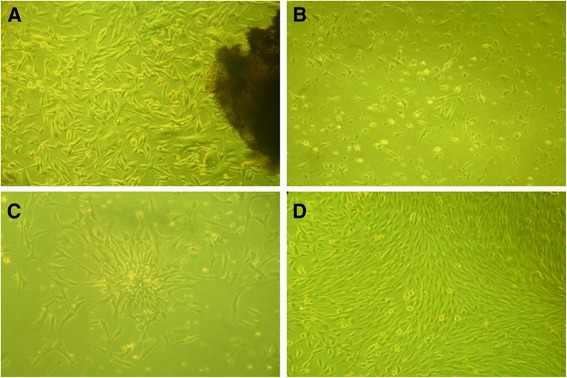
**Culture of periodontal ligament (PDL) cells from rabbits. (A)** The PDL cells digested from rabbit periodontal tissue were cultured for 10 days under a light microscope. **(B)** The morphology of the primary PDL cells under a light microscope. **(C)** Cell clone was established after 15 days of culture. **(D)** The isolated PDL cells grew vigorously after subculture. Magnification: 100 ×.

To isolate PDL cells, single-cell suspension was obtained by enzymatic digestion and placed into the culture medium. Primary PDL cells cultured by the tissue explant culture method were adherent after 2 hours of culture, and the culture reached confluence 6 hours later. After 10 days in culture, primary PDL cells reached the edge of the tissue block (Figure 2A). The isolated cells had typical fibroblastic morphology, a spindle shape with extending cytoplasmic processes (Figure 2B).

**Figure 3:**
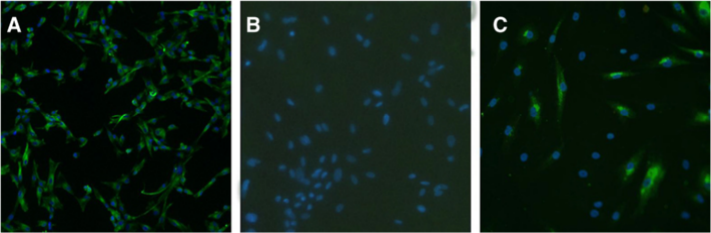
**Immunocytochemical analysis of periodontal ligament stem cells. (A)** Positive staining for vimentin. **(B)** Negative staining for keratin. **(C)** STOR-1 was partially positively stained. DAPI (4′,6-diamidino-2-phenylindole) was used for staining nuclei. Magnification: 100 ×.

To obtain PDLSCs and determine the proliferation and clonogenic potential of the cells, we performed a limiting dilution assay using first-passage PDL cells. Clones were evident after 15 days of culture, with a lot of small fusiform or triangular cells arranged closely (Figure 2C). The PDL cells grew vigorously after subculture (Figure 2D). Immunofluorescence analysis of PDLSCs showed positive staining for vimentin but negative staining for keratin, confirming their mesodermal origin (Figure 3A and B). PDLSCs stained positively for STRO-1, confirming their stromal stem cell status (Figure 3C).

### Osteogenic and adipogenic differentiation of periodontal ligament stem cells

**Figure 4:**
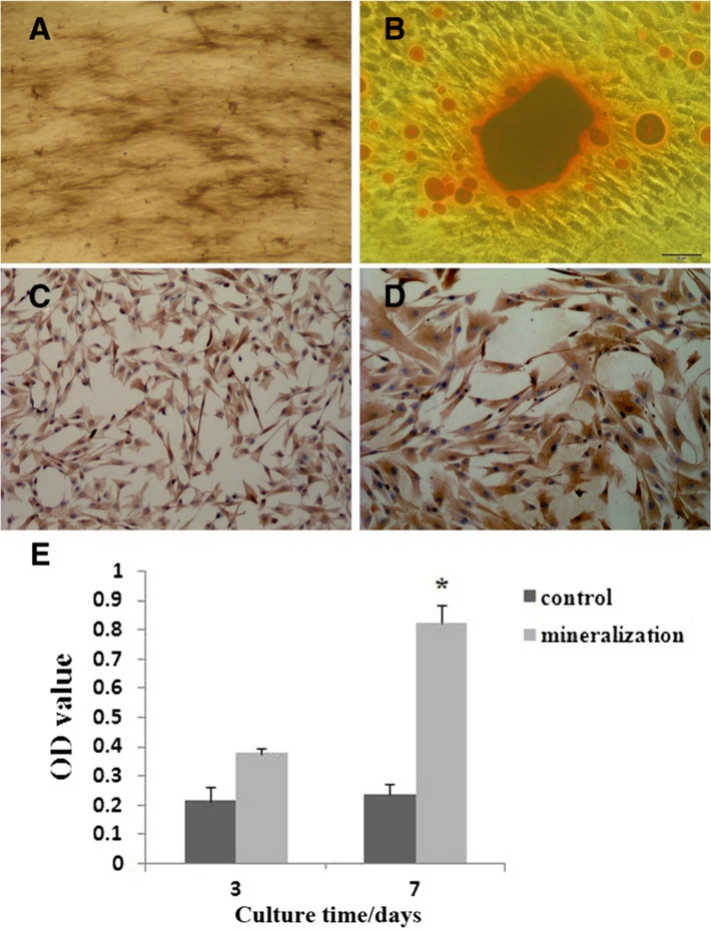
***In vitro*** **osteogenesis of periodontal ligament stem cells. (A)** Cells grown in osteogenic media stained positively for alkaline phosphatase (ALP) **(A)** and Alizarin red **(B)** after 28 days of culture and for osteocalcin **(C)** and type I collagen **(D)** using immunocytochemistry after 21 days of culture. **(E)** ALP activities in osteogenic condition increased significantly at day 7 when compared with that at day 3, but there was no significant difference in ALP activities at days 3 and 7 in the control group. *P <0.05 versus control group. Magnifications: 100× **(A, C)** and 200× **(B, D)**. OD, optical density.

**Figure 5:**
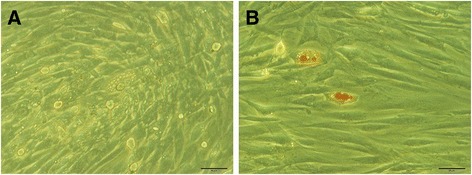
***In vitro*** **adipogenesis of periodontal ligament stem cells.** Cells grown in adipogenic media demonstrate the presence of fat vacuoles under a light microscope **(A)** and abundant fat vacuoles by Oil red O staining **(B)** after 6 weeks of culture. Magnification: 200 ×.

To assess the multipotent capability of PDLSCs, cells were cultured in osteogenic and adipogenic conditions to induce differentiation of PDLSCs. Differentiation into osteoblasts was confirmed by intense staining for ALP (Figure 4A), Alizarin red (Figure 4B), OCN (Figure 4C), and type I collagen (Figure 4D). Furthermore, ALP activities varied during culture period (7 days) (Figure 4E). ALP activities increased dramatically at day 7 compared with those at day 3. There was no significant difference of ALP activities at day 3 (*P* >0.05). ALP activities of the cells in osteogenic condition were significantly higher than those of the control group at day 7 (*P* <0.05). Differentiation into adipocytes was confirmed by the presence of fat vacuoles under a light microscope and Oil red O-positive lipid droplets in PDLSCs after 6 weeks of induction (Figure 5A and B).

### Transfection of periodontal ligament stem cells

**Figure 6:**
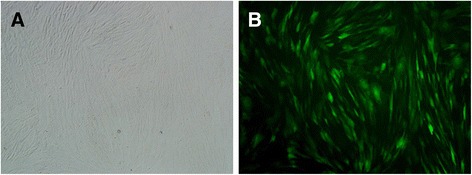
**Images of periodontal ligament stem cells transfected with pLENTi6.3/V5-hOPG-IRES-EGFP plasmid. (A)** Bright field image. **(B)** Fluorescent microscope image. Magnification: 100×. EGFP, enhanced green fluorescent protein; hOPG, human osteoprotegerin; IRES, internal ribosome entry site.

Lentiviral vector pLENTi6.3/V5-hOPG-IRES-EGFP containing hOPG cDNA was successfully constructed and transfected into PDLSCs. Within 48 hours after lentiviral transfection, obvious green fluorescence expression can be observed under fluorescence microscope (Figure 6).

**Figure 7:**
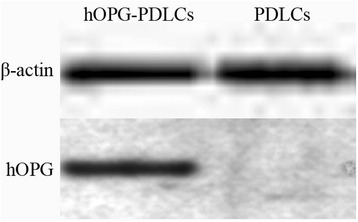
**Western blot analysis of the expressions of human osteoprotegerin** **(hOPG) in transfection and non-transfection groups.** Results show that hOPG-EGFP fusion gene was co-expressed, suggesting that hOPG protein was successfully expressed in periodontal ligament stem cells (PDLSCs). hOPG-PDLCs: PDLSCs transfected with pLENTi6.3/V5-hOPG-IRES-EGFP plasmid; PDLCs: control sample group: non-transfected PDLSCs. eGFP, enhanced green fluorescent protein.

To investigate the effects of transfection of lentiviral vector pLENTi6.3/V5-hOPG-IRES-EGFP on the expression of hOPG in PDLSCs, Q-PCR and Western blot were used to quantify the mRNA and protein levels of hOPG in the PDLSCs with or without OPG transfection, after 48 hours of culture. As shown in Table 2, the relative mRNA level of OPG was significantly increased in hOPG-transfected cells by 3,311.65-fold compared with the mRNA expression levels of hOPG in non-transfected cells. Western blot analysis results of pLENTi6.3/V5-hOPG-IRES-EGFP transfected PDLSCs show that a band at 50 to 64 KD was observed at 72 hours after transfection (Figure 7), and its size was consistent with the size of hOPG fusion protein (60 kDa), indicating that hOPG protein was expressed in PDLSCs.

**Table 2:** **Relative mRNA levels of human osteoprotegerin in the transfection and non-transfection groups**

**Group**	**Human osteoprotegerin mRNA (2** ^**−△△Ct**^ **)**
Non-transfection	1
Transfection	3311.65

### Scanning electron microscopy

**Figure 8:**
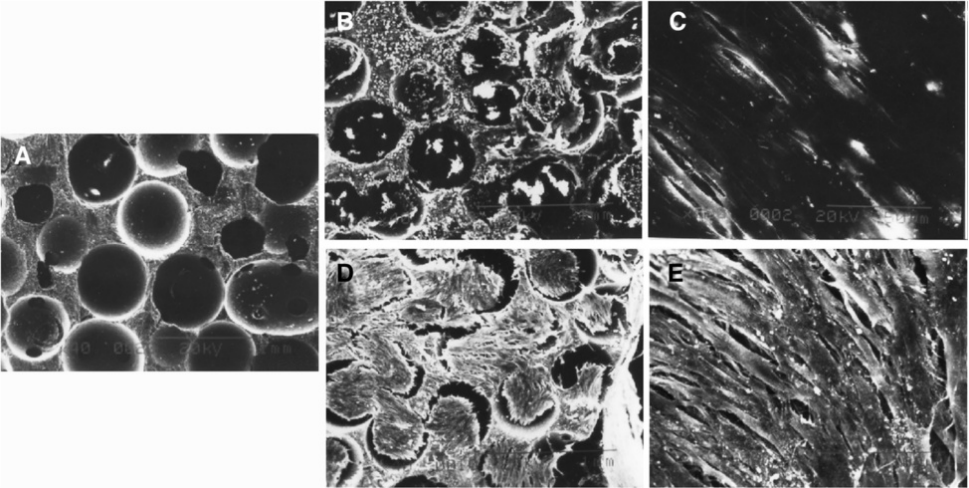
**Images of β-TCP and PDLSCs/β-TCP constructs with or without hOPG transfection were evaluated by SEM. (A)** Image of β-TCP under SEM. **(B, C)** The PDLSCs adhered and extended on β-TCP surface after 2 days of culture. **(D, E)** The PDLSCs grew in number to link flakiness on the β-TCP surface and in the pore of the scaffold material after 7 days of culture. Magnifications: 40× **(A, B, D)** and 400× **(C, E)**. β-TCP, beta-tricalcium phosphate; hOPG, human osteoprotegerin; PDLSC, periodontal ligament stem cell; SEM, scanning electron microscope.

The images of β-TCP and PDLSCs/β-TCP constructs with or without hOPG transfection were evaluated by SEM, and results are presented in Figure 8. Under SEM, β-TCP blocks showed three-dimensional open, network structure with continuous void volume connecting adjacent pores (Figure 8A). The PDLSCs with or without hOPG transfection were impregnated onto β-TCP porous scaffolds. After 2 days of culture, the PDLSCs could be seen adhered and extended on the β-TCP surface and in the pore of the scaffold material (Figure 8B and C). After 7 days of culture, a large number of PDLSCs could be seen adhered and significantly grown in number to link flakiness on the surface and in the pore of the scaffold material, and there were many filarious extracellular matrices (Figure 8D and E). No significant difference in growth of PDLSCs on β-TCP was observed between the transfection group and the non-transfection group.

### Histological analysis of *in vivo* studies

**Figure 9:**
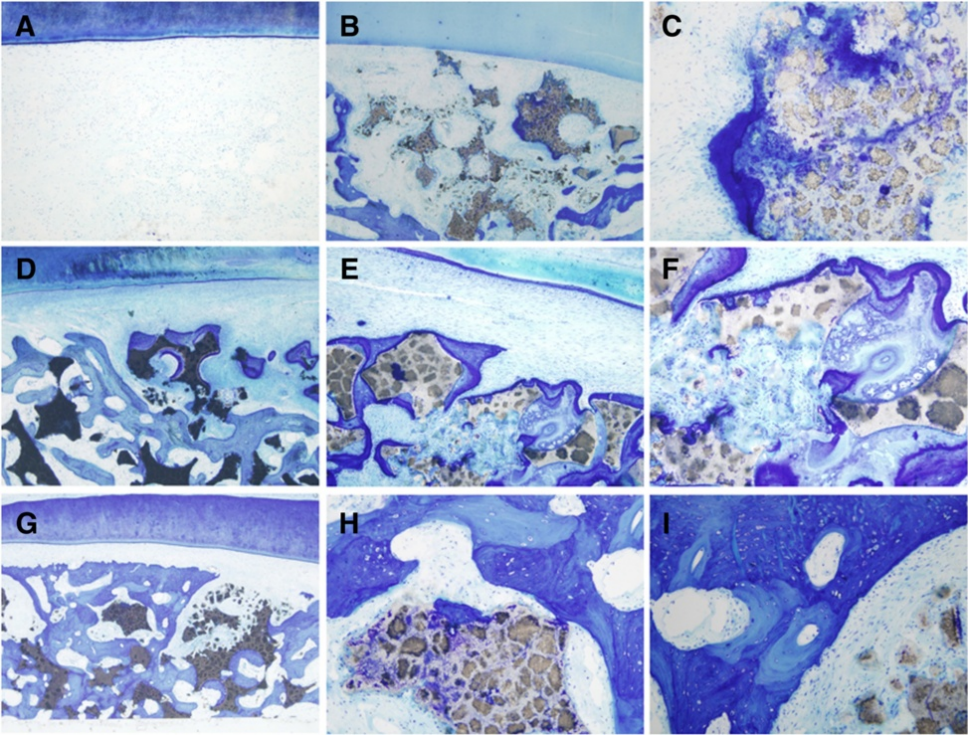
**Histological sections stained by Toluidine blue of the alveolar bone defects at 12 weeks after implantation. (A)** No bone regeneration was detected at the alveolar bone control group. A small amount of new bone could be seen in the β-TCP group, with some osteoid formation in the periphery and center of the β-TCP scaffold **(B, C)**. The PDLSCs/β-TCP group showed more new alveolar bone formation, with numerous small bone trusses or trabeculae interconnected with each other **(D-F)**. A maximal and robust bone formation was presented in the hOPG-PDLSCs/β-TCP group. Osteoblastic cells were lining the surface of newly formed bone. Osseous maturation had increased, with osteon formation and increased bone density **(G-I)**. Magnifications: 40× **(A, B, D, G)**, 100× **(E)**, and 400× **(C, F, H, I)**. β-TCP, beta-tricalcium phosphate; hOPG, human osteoprotegerin; PDLSC, periodontal ligament stem cell.

The PDLSCs with or without hOPG transfection were impregnated onto β-TCP porous scaffolds, and cell/scaffold constructs were implanted into alveolar bone defects of rabbits. After 12 weeks, histological sections of alveolar bone defects were analyzed for new bone formation. Toluidine blue staining showed that no bone regeneration was detected at the alveolar bone control group, and there was remarkable ingrowth of muscle fibers into the defect (Figure 9A). A small amount of new bone could be seen in the β-TCP group, with some osteoid formation in the periphery and center of the β-TCP scaffold (Figure 9B and C). The PDLSCs/β-TCP group showed more new alveolar bone formation, with numerous small bone trusses or trabeculae interconnected with each other (Figure 9D, E, and F). A maximal and robust bone formation was presented in the hOPG-PDLSCs/β-TCP group. Osteoblastic cells were lining the surface of newly formed bone. Osseous maturation had increased, with osteon formation and increased bone density (Figure 9G, H, and I).

### Circularly polarized light microscopy

**Figure 10:**
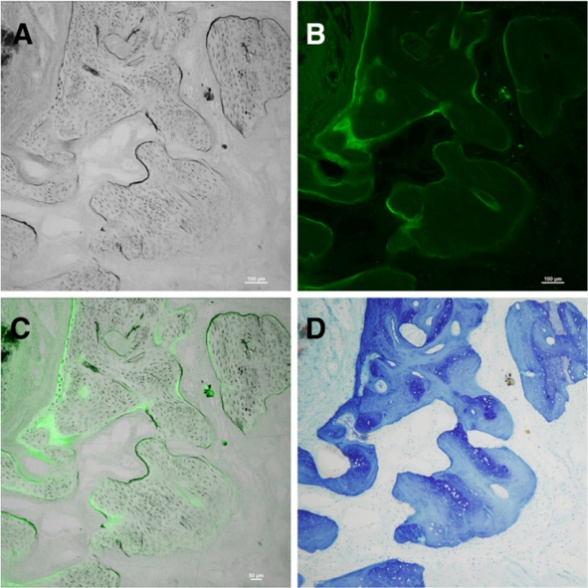
**New bone regeneration assessment of hOPG-transfected PDLSCs/β-TCP constructs by circularly polarized light microscopy. (A)** The osteoblasts and bone lacunas were clearly observed in the newly formed bone by using light microscopy. **(B)** At the same visual field, the hOPG-GFP-transfected PDLSCs were observed by using a fluorescence microscope. **(C)** When images **(A)** and **(B)** were overlapped, the positions of some GFP^+^ cells were superposed with the osteoblasts and bone lacunas. The newly formed bone was also clearly observed in Toluidine blue-stained tissue sections **(D)**. Magnification: 100×. β-TCP, beta-tricalcium phosphate; GFP, green fluorescent protein; hOPG, human osteoprotegerin; PDLSC, periodontal ligament stem cell.

To assess whether the hOPG-transfected PDLSCs/β-TCP complex had implanted into the bone defects and had differentiated into osteoblasts, periodontal tissue samples from the hOPG-PDLSCs/β-TCP group were first observed by using light microscopy to identify the newly formed bone (Figure 10A). In the same visual field, the hOPG-GFP-transfected PDLSCs were then observed by fluorescence microscopy (Figure 10B). When the images shown in Figure 10A and B were overlapped, the positions of some GFP^+^ cells were superposed with the osteoblasts and bone lacunas (Figure 10C). The newly formed bone was also clearly observed in Toluidine blue-stained tissue sections (Figure 10D). All of these results indicated that the GFP^+^ cells derived from *ex vivo*-expanded PDLSCs had differentiated into osteoblasts *in vivo*.

### Histomorphometric analysis

**Figure 11:**
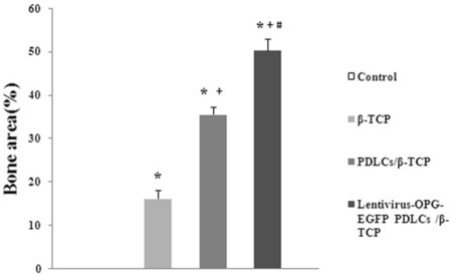
**Histomorphometric findings of new bone formation (percentage) in the constructs after 12 weeks of implantation (n = 5).** *P <0.05 versus the control group; ^+^ *P* <0.05 versus the β-TCP group; ^#^ *P* <0.05 versus the PDLSCs/β-TCP group. β-TCP, beta-tricalcium phosphate; EGFP, enhanced green fluorescent protein; OPG, osteoprotegerin; PDLSC, periodontal ligament stem cell.

The results of histomorphometric analysis are summarized in Figure 11. The percentage of new bone formed area in the hOPG-transfected PDLSCs/β-TCP group was significantly higher than that formed in the PDLSCs/β-TCP group, the β-TCP group, and the control group. This value for hOPG-transfected PDLSCs/β-TCP was significantly higher than that for PDLSCs/β-TCP and β-TCP (*P* <0.05), and the value for PDLSCs/β-TCP was significantly higher than that for β-TCP (*P* <0.05).

## Discussion

Alveolar bone destruction is a common hallmark of human periodontitis, a heterogeneous disease in etiology, and is one of the major causes of tooth loss in humans [27]. There appears to be an urgent need for the concept of tissue and bone regeneration around implants because of the increasing need to use dental implants for the growing population of patients and to enhance their function to simulate normal tooth physiology and proprioception [6].

Tissue-engineering approaches, involving the utilization of *in vitro*-expanded cells with regenerative capacity and their delivery to the appropriate sites using biomaterial scaffolds, have been proposed as promising alternatives to conventional treatments [6,28]. Stem cell-based regenerative approaches combined with the usage of emerging biomaterials are entering a new era in periodontal regeneration. PDL cells, *in vitro*-expanded cells in sufficient quantities and possessing the potential to regenerate alveolar bone and cementum, could be used with appropriate biomaterials to engineer living tissues *in vitro* for subsequent transplantation into defect sites [29]. Meanwhile, the utilization of gene therapy to sustain the release and bioavailability of osteogenic growth factors also offers potential for periodontal tissue-engineering and regeneration applications [30]. In this study, we demonstrated that gene-modified rabbit PDLSCs expressing hOPG combined with biodegradable β-TCP scaffold achieved an earlier mineralization and more bone formation when compared with β-TCP and PDLSCs-β-TCP, which may help to ensure the reconstruction of alveolar bone defect.

MSCs are important cell resources for periodontal tissue engineering; among them, PDLSCs are a candidate cell source for periodontal regeneration and have proven to be the ideal seeding cells for gene therapy in periodontal tissue engineering [31]. Therefore, PDLSCs from rabbit were used for periodontal tissue engineering in this study. PDLSCs were isolated by a limiting dilution method and were characterized by immunofluorescence analysis. The validations of PDLSCs are important for cytotherapeutic uses. For these reasons, we have associated STRO-1, vimentin, and keratin expressions to isolate a population of MSCs. The results showed that PDLSCs were positively stained for STRO-1 and vimentin and negatively for keratin, confirming their mesodermal origin and stromal stem cell status. A significant number of studies have been conducted to evaluate the regenerative capacity of PDLSCs *in vivo* and *in vitro*, and PDLSCs were demonstrated to differentiate into many pathways under defined culture conditions [32]. Our study here showed that isolated PDLSCs represent an approachable niche of stem cells when cultured in conditioned media and are able to extensively proliferate and differentiate into several cytotypes, mainly osteoblasts, expressing osteogenic markers ALP, OCN, and type I collagen as well as forming mineralized nodules *in vitro*. Our single-colony-derived cells were also found to be capable of differentiating into other cell lineages, such as adipocytes, as demonstrated by the presence of fat vacuoles and Oil red O-positive lipid droplets after 28 days of culture, confirming their pluripotent stem origin. All of these results further confirmed that the isolated cell population constitutes a large, ideal source of osteoblasts, and the *in vitro*-expanded PDLSCs with regenerative capacity are suitable for bone regeneration, transplantation, and tissue-based therapies.

The morphogenesis and remodeling of bone depend on the integrated activity of osteoblasts that form bone and osteoclasts that resorb bone [33]. OPG (or osteoclastogenesis inhibitory factor, OCIF), a naturally occurring secreted protein with homology to members of the TNF receptor family, is a soluble decoy receptor for RANKL, a critical osteoclast differentiation factor that selectively inhibited osteoclast differentiation and function *in vitro* and *in vivo* [34,35]. Administration of OPG *in vivo* has been shown to inhibit osteoclastogenesis and associated bone resorption and blocks the pathological increase in osteoclast numbers and activity seen in animal models that mimic osteopenic disorders in humans [36]. *In vivo* inhibition of OPG function with OPG ligand diminished alveolar bone destruction and reduces the number of periodontal osteoclasts after microbial challenge [19]. Systemic administration of OPG interfering with RANKL abrogated periodontal bone resorption in the rat model, and treatment with kaliotoxin, a scorpion venom potassium channel inhibitor, resulted in decreased RANKL expression, diminished induction of RANKL-dependent osteoclastogenesis, and abrogation of bone resorption [37]. However, the latter was also reported to be abrogated by OPG fusion protein [38]. Therefore, hOPG may promise tremendous hope for potential clinical use in the management of human periodontal disease.

However, in human clinical trials, direct application of OPG may be limited in action. A major problem that needed to be overcome is how to localize the delivery of this short half-lived factor to target cells since large doses of this factor in treatment can result in adverse side effects [39,40]. Thus, maintaining systemically stable, therapeutic levels of OPG will be critical for preventing osteolytic bone damage. To address this problem and to acquire localized, continuous expression of OPG in target cells and tissue, increasing attention has been focused on the use of gene therapy technique [17,41]. The use of gene therapy technique is more advantages in the local expression than continuously injecting OPG or recombinant protein, and appears to be a highly promising adjuvant therapy to this end.

The efficient delivery of growth-promoting genes locally in a sustained manner is important for effective tissue regenerations, and the efficacy of a combinatorial gene and cell therapeutic approach also depends on transgene expression vector. Reporter genes, like eGFP, a chromophore-containing protein, have generally been used for the gene expression and regulation to determine the efficiency of gene vector delivery [20]. After direct comparison of different gene therapy vectors in the stem cell preparation, McMahon *et al*. [42] indicated that lentivirus was the most effective vector for stem cell transduction and that high and moderate levels of cell transduction using lentivirus vectors did not affect the ability of the cells to differentiate down the adipogenic pathway. Corroborating these results, Wen *et al*. [31] (2012) demonstrated that lentiviral vector with eGFP was an appropriate expression vector system and that human PDLSCs were ideal seeding cells for gene therapy in periodontal tissue engineering. Therefore, in this study, lentiviral vector pLenti6.3/V5-DEST containing eGPF and hOPG was constructed by using Gateway cloning technology for gene therapy, and the expressions of hOPG were shown to be significantly enhanced in both mRNA and protein levels after transfection into rabbit PDLSCs.

In addition to the preparation of PDLSCs and construction of hOPG-engineered lentiviral vector, the development of suitable bioactive three-dimensional scaffolds for promotion of cellular proliferation and differentiation is critical in periodontal tissue engineering. β-TCP has been shown to have good biocompatibility and osteoconductivity in both animal experiments and clinical settings [8,10]. β-TCP material was therefore used in this study, and rabbit PDLSCs with or without engineered hOPG were seeding on β-TCP scaffolds. SEM results showed that β-TCP blocks were three-dimensional open, network structure with continuous void volume connecting adjacent pores. The PDLSCs with or without hOPG transfection were impregnated onto β-TCP porous scaffolds. After 7 days of culture, a large number of PDLSCs could be seen adhered and significantly grown in number to link flakiness on the surface and in the pore of scaffold material, and there were many filarious extracellular matrices. Interestingly, no significant difference in growth of PDLSCs on β-TCP was observed between transfection and non-transfection groups. These results demonstrated that β-TCP supported the attachment, growth, and differentiation of PDLSCs and possessed good biocompatibility.

To further assess the hOPG-engineered PDLSCs-β-TCP complex on alveolar bone regeneration, we implanted β-TCP with or without PDLSCs as well as hOPG-engineered PDLSCs-β-TCP complex into the alveolar bone defect rabbit models. After 12 weeks, histological sections of alveolar bone defects were analyzed for new bone formation. Toluidine blue staining showed that hOPG-engineered PDLSCs-β-TCP tissue-engineered complex had an earlier mineralization and more bone formation inside the scaffold than control, β-TCP, and PDLSCs-β-TCP. The results indicated that alveolar bone repair was significantly enhanced when PDLSCs cultured on β-TCP were transfected with exogenous hOPG *in vivo*. Several studies have also demonstrated, even without the artificial extracellular matrix substitutes and the three-dimensional environment used for cell culture, that bone repair is significantly enhanced when OPG delivery is supplemented with osteogenic cell populations [17,41]. MSC genes modified with OPG were also reported to be able to reverse osteoclast activation in a xenogeneic model of multiple myeloma [38]. All of these results suggested that the local stem cell niche is a limiting factor and that the implanted scaffold with PDLSCs engineered by hOPG could create enough extracellular cell matrix for adequate healing.

Meanwhile, circularly polarized light microscopy results further confirmed the success of hOPG-transfected PDLSCs-β-TCP complex implantation into rabbit bone defects of rabbits and osteoblast differentiation *in vivo*. The new bone formation in defects was evaluated by using histomorphometric analysis. Results showed that the percentage of new bone formed area in the hOPG-transfected PDLSCs-β-TCP group was significantly higher than that in the PDLSCs-β-TCP group, the β-TCP group, and the control group, suggesting that hOPG-transfected PDLSCs-β-TCP complex could very well integrate into host alveolar bone and could be used as a promising tissue-engineering technology for alveolar bone regeneration.

## Conclusions

The present study demonstrated the feasibility of using β-TCP scaffolds for primary PDLSC culture and expression of hOPG gene *in vitro* and *in vivo*, and the β-TCP scaffolds containing hOPG-engineered PDLSCs exhibited the highest proliferation rate and an earlier mineralization and more bone formation inside the scaffold. The above results suggested the potential of systemic hOPG gene therapy in combination with PDLSC tissue engineering as a good candidate in periodontal tissue engineering for alveolar bone regeneration.

## Abbreviations

β-TCP: beta-tricalcium phosphate
ALP: alkaline phosphatase
DMEM: Dulbecco’s modified Eagle’s medium
EGFP: enhanced green fluorescent protein
FBS: fasting blood sugar
hOPG: human osteoprotegerin
MSC: mesenchymal stem cell
MTT: 3-(4,5-dimethylthiazol-2-Yl)-2,5-diphenyltetrazolium bromide
OPG: osteoprotegerin
PBS: phosphate-buffered saline
PDL: periodontal ligament
PDLSC: periodontal ligament stem cell
PLCSC: peripheral and limbal corneal stromal cell
Q-PCR: quantitative real-time reverse transcription-polymerase chain reaction
RANKL: receptor activator of nuclear factor-kappa-B ligand
SEM: scanning electron microscope
TNF: tumor necrosis factor
